# 2017 ISCB Accomplishment by a Senior Scientist Award given to Pavel Pevzner

**DOI:** 10.1371/journal.pcbi.1005561

**Published:** 2017-06-30

**Authors:** Christiana N. Fogg, Diane E. Kovats, Bonnie Berger

**Affiliations:** 1 Freelance science writer, Kensington, Maryland, United States of America; 2 Department of Mathematics, MIT, Cambridge, Massachusetts, United States of America; 3 International Society for Computational Biology, Bethesda, Maryland, United States of America; 4 Computer Science and Artificial Intelligence Laboratory, MIT, Cambridge, Massachusetts, United States of America

**Figure:**
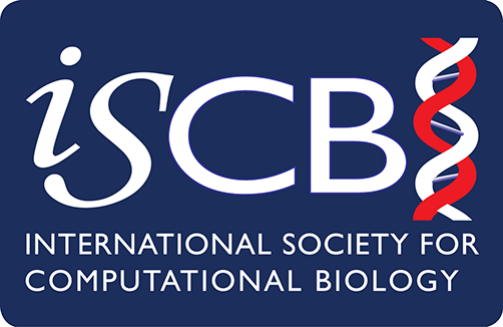


The International Society for Computational Biology (ISCB) recognizes an established scientist each year with the Accomplishment by a Senior Scientist Award for significant contributions he or she has made to the field. This award honors scientists who have contributed to the advancement of computational biology and bioinformatics through their research, service, and education work. Pavel Pevzner, PhD, Ronald R. Taylor Professor of Computer Science and Director of the NIH Center for Computational Mass Spectrometry at University of California, San Diego, has been selected as the winner of the 2017 Accomplishment by a Senior Scientist Award.

The ISCB awards committee, chaired by Dr. Bonnie Berger of the Massachusetts Institute of Technology, selected Pevzner as the 2017 winner. Pevzner will receive his award and deliver a keynote address at the 2017 Intelligent Systems for Molecular Biology-European Conference on Computational Biology joint meeting (ISMB/ECCB 2017) held in Prague, Czech Republic, from July 21–25, 2017. ISMB/ECCB is a biennial joint meeting that brings together leading scientists in computational biology and bioinformatics from around the globe.

## Pavel Pevzner: In search of life’s perfect puzzles

**Fig 1:**
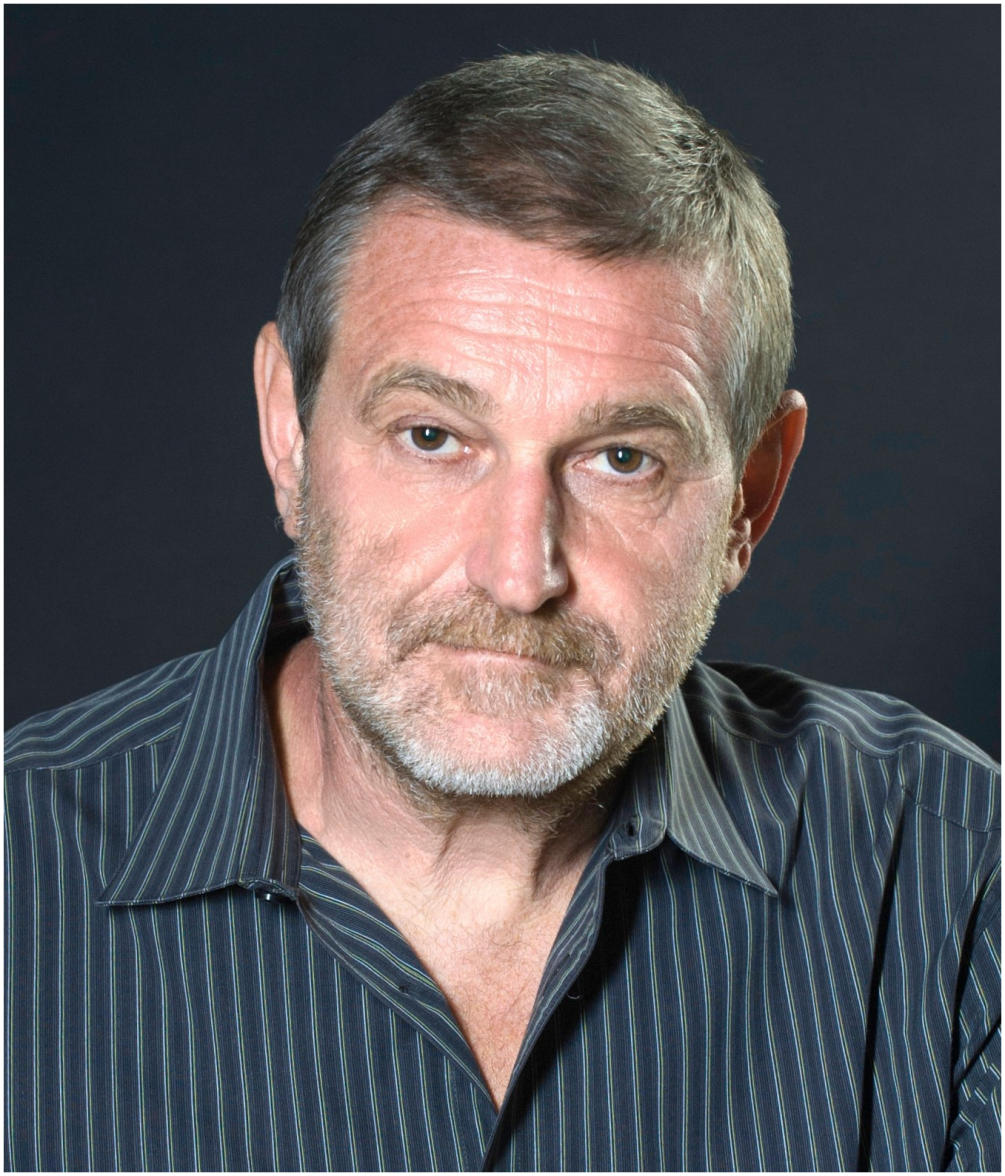
Pavel Pevzner.

Pavel Pevzner (Fig 1) was born in Kursk, Russia, and spent his childhood in the city of Murom, which was a hub of the Soviet electronics industry. His father was an electrical engineer and his mother was a teacher, but he admits that his early education got off to a rocky start. He described himself as a poor student who was more interested in having fun, but around age 10, he grew more interested in books [1]. Pevzner’s interest and abilities in mathematics were soon recognized, and at age 14 he was sent to the Boarding High School at Moscow State University, founded by world-renowned mathematician Andrey Kolmogorov, for children gifted in math and physics. In spite of Pevzner’s rigorous high school math studies, he had difficulty getting into Moscow State University because of the anti-Semitic admission policies aimed at ethnic Jews [2].

As an undergraduate, Pevzner studied at the Russian Institute of Railway Engineers, which was known for its applied mathematics program. He did well throughout his course of study and published a number of papers on discrete mathematics as an undergraduate. In 1985, he joined a bioinformatics lab at Institute of Genetics of Microorganisms VNIIGENETIKA and received his PhD in mathematics and physics in 1988 from the Moscow Institute of Physics and Technology.

Pevzner was completing his graduate work during the era of “perestroika” and “glastnost,” and for the first time in decades, scientists were being granted permission to travel abroad and were even told that the government would pay for their travels. Pevzner jumped on this opportunity and notified the Russian government that he wanted to work with Mike Waterman at the University of Southern California (USC), a pioneer in the field of bioinformatics and 2006 ISCB Accomplishments by a Senior Scientist Award winner. In 1989, Pevzner reached out to Waterman personally, not quite trusting that his request to the government would be enough to facilitate his travels (it was never granted). During their correspondence, Waterman sent him an open problem, which Pevzner ended up solving. Waterman was surprised that someone had solved this problem and eventually invited him to pursue postdoctoral work in his lab. Pevzner spent 2 years as a postdoc with Waterman at USC.

In 1992, Pevzner established himself as an independent researcher and started his lab in the Department of Computer Science at Pennsylvania State University. He returned to USC 3 years later as a professor in the Department of Mathematics. In 2000, he moved to San Diego and was named Ronald R. Taylor Chair Professor of Computer Science at the University of California, San Diego (UCSD). In 2006, he was named a Howard Hughes Medical Institute Professor.

Pevzner’s research interests span the field of bioinformatics, and his work has been guided by applying algorithmic ideas to bioinformatics problems. Pevzner has made significant contributions to a wide array of subfields, including genome assembly, understanding how genome rearrangements influence evolution, and developing new algorithms for sequencing antibodies and antibiotics using mass spectrometry. He continues to be fascinated and amazed by scientific discovery, and he explained, “The most surprising thing for me was a realization that dominant biological theory often falls apart when new data and new methods to analyze them become available. Three times in my career, I had to refute the biological theories that I worked on: the Master Alu theory of repeat evolution, the NME theory that connects the N-terminal methionine excision and protein half-lives, and the Random Breakage Theory of chromosome evolution.”

In this era of abundant genome data, Pevzner is currently interested in reconstructing the detailed evolution of the human genome, down to each new repeat and rearrangement that affected the genome, by using hundreds of primate genomes that will be sequenced in the near future. In a more practical domain, Pevzner’s other goal is to develop a computational approach to antibiotics discovery, a problem of great importance due to emerging antibiotics resistance. He describes antibiotics discovery as one of the last bastions of modern biology that remains barely touched by bioinformatics.

Beyond Pevzner’s academic contributions, he has served the computational biology community in many ways. He helped launch RECOMB (Conference in Research in Computational Molecular Biology) 17 years ago, together with Waterman and Sorin Istrail. He has served as a member of numerous editorial boards in the fields of computational biology and computer science.

Throughout his career, Pevzner has mentored many students; 22 of his former graduate and postdoctoral trainees are now professors at various universities. He has seen significant changes to the way he teaches his undergraduate courses along the way. He explained, “The way I teach my undergraduates has completely changed: I haven’t given a traditional classroom lecture in 3 years now. I feel that traditional 1,000-year-old educational technology (classroom lecture) is coming to its end and will soon be substituted by more efficient “intelligent tutoring systems.” My goal in recent years was to develop the first such system for bioinformatics that will be launched in spring 2017 on edX.”

Pevzner is humbled by his selection as the winner of the 2017 ISCB Accomplishment by a Senior Scientist Award—he acknowledges that he did not earn this recognition alone. He said, “This award really goes to my teachers and my students and postdocs over the last quarter of a century.”
